# Stability of a surrogate African swine fever-like algal virus in corn- and soybean-based feed ingredients during extended storage and *in vitro* digestion processes

**DOI:** 10.3389/fvets.2024.1498977

**Published:** 2024-11-27

**Authors:** Gerald C. Shurson, Christian D. Ramirez-Camba, Pedro E. Urriola, Declan C. Schroeder

**Affiliations:** ^1^Department of Animal Science, College of Food, Agricultural and Natural Resource Sciences, University of Minnesota, St. Paul, MN, United States; ^2^Department of Veterinary Population Medicine, College of Veterinary Medicine, University of Minnesota, St. Paul, MN, United States

**Keywords:** African swine fever virus, corn-based ingredients, *Emiliania huxleyi* virus, extended storage, *in vitro* digestibility, soybean-based ingredients, viability PCR

## Abstract

Prevention of transmission of African swine fever virus (ASFV) through contaminated feed ingredients and complete feed is an important component of biosecurity protocols for global feed supply chains. Use of extended storage times for feed ingredients has become a popular and emerging mitigation strategy that may allow partial inactivation of ASFV before manufacturing swine feeds. However, the effectiveness of this strategy is unclear because limited studies have been conducted using diverse methodologies and insufficiently sensitive measures of virus viability of only a few types of feed matrices. Therefore, interpretation of results from these studies has made providing prudent recommendations difficult. Furthermore, although a few studies have shown that feed is a plausible route of transmission of ASFV to pigs, there are conflicting findings on the infectivity of ASFV that may be present in feed, which may be related to the extent that ASFV is degraded in the pig’s digestive system after it is consumed. Therefore, the objectives of this study were to use a surrogate ASFV-like algal virus (*Emiliania huxleyi*; EhV) to determine stability in corn- and soybean-based feed ingredients and complete feed during a 120-day storage period at temperatures up to 34°C, and EhV survival in various feed matrices during three stages of an *in vitro* digestion process. Results indicated that inoculating corn- and soybean-based feed ingredients and complete feed with EhV and storing them at 4°C, 24°C, or 34°C for up to 120 days did not result in the complete inactivation of EhV in any of these matrices. Because EhV has similar environmental and thermal resilience to ASFV, these results indicate that both viruses can maintain viability in various feed matrices during long-term storage and suggest that extending storage time up to 120 days is not an effective mitigation practice against ASFV. We also determined that between approximately 5- to more than 7-log (99.999 to 99.99999%) reductions in EhV in various feed matrices occur during the entire *in vitro* digestion and fermentation process. These reductions appear to be correlated with the chemical composition of the matrices, potentially explaining inconsistencies in ASFV infection when pigs consume infectious doses of contaminated feed.

## 1 Introduction

African swine fever virus (ASFV) continues to infect hundreds of thousands of pigs in numerous countries worldwide, causing enormous economic losses and significantly increasing the environmental footprint of pork production systems (1). Although some progress has been made in vaccine development, it has yet to become a viable disease prevention and control strategy (2–5). Furthermore, there are no treatments to control ASFV, but some antiviral feed additives have been shown to be effective for partially inactivating ASFV in various feed ingredients and complete feeds under experimental conditions (6, 7). As a result, the most prudent course of action to prevent the spread and subsequent infection is through strict biosecurity protocols (8).

Although the likelihood of transmission and subsequent ASFV infection through feed ingredients and complete feeds is low relative to direct exposure to infected pigs, carcasses, tissues, and body fluids (9, 10), it remains a plausible route that has generated considerable research during the past few years (11–13). Unfortunately, there is no standardized analysis or monitoring system to determine the potential presence, concentration, and stability of ASFV in contaminated ingredients (14). As a result, the use of extended storage times has become a popular and emerging approach to partially inactivate ASFV and other swine viruses that may be present in feed ingredients (14). However, the methodologies used to determine the effectiveness of storage time and temperature have been limited to only a few ingredients (i.e., soybean meal) and have led to highly variable results that are difficult to interpret (13, 14). Lack of sensitivity to detect viable ASFV has contributed to different interpretation of results. Viable ASFV is defined as structurally intact virus particles that can still be infectious when taken up via the macropinocytotic infection route (15). Furthermore, ASFV is much more thermally resilient than previous studies (16) have shown when viability PCR is used (15). We have developed a surrogate assay using an ASFV-like algal virus (*Emiliania huxleyi*; EhV) to simulate ASFV in feed matrices (15, 17). We have also developed a modified *in vitro* digestibility procedure to evaluate the digestion and fermentation of various types of feed ingredients in pigs (18).

Compared with other feed ingredients, soybean meal appears to have unique properties that enable ASFV survival (19, 20) under simulated conditions of a 30-day transoceanic transport and enables ASFV to survive for many months of storage at temperatures up to 35°C (21). Similarly, Palowski et al. (17) showed that, when using EhV as a surrogate for ASFV, no degradation was detected in conventional and organic soybean meal and complete feed samples after a 23-day truck transportation event. Furthermore, Palowski et al. (17) also showed that the majority of EhV remains bound to soybean meal after extraction for PCR or bioassay analysis, which makes soybean meal an ingredient of potential concern for transmission of ASFV. In addition, although corn is the predominant ingredient used in swine diets around the world, it has not been evaluated in ASFV storage studies to the same extent as soybean meal, nor have other types of corn co-products used in swine diets been evaluated.

Moreover, there are conflicting findings on the infectivity of ASFV post-extraction from feed, which may be related to the extent that ASFV is degraded in the pig’s digestive system after it is consumed. Two studies have shown that feed and water can be routes of ASFV transmission. Niederwerder et al. (22) determined the minimum infectious dose of ASFV in feed to be 10^4^ TCID_50_ with a minimum dose of 10^0^ TCID_50_ for liquid. However, Blázquez et al. (23) reported that the minimum infectious dose of ASFV is greater than 10^5^ because feeding diets inoculated with 10^5^ TCID_50_ of ASFV in liquid plasma for 14 consecutive days failed to cause disease. Reasons for these conflicting results are unclear but may be related to different feed constituents used in each study or due to virus survival during the various stages of the digestive process. No studies have attempted to determine the fate of ASFV-contaminated feed ingredients in the pig gastrointestinal tract during the digestion and fermentation process. Therefore, the objectives of this study were to (1) evaluate survival of EhV, as a surrogate for ASFV, in corn- and soybean-based ingredients and complete feed at different storage temperatures up to 34°C during a 120-day storage period, and (2) determine the survival of EhV in corn- and soybean-based ingredients and complete feed during the simulated *in vitro* hydrolysis and fermentation stages of the digestive process in pigs.

## 2 Materials and methods

### 2.1 Sample collection

Representative samples of dehulled, solvent-extracted soybean meal, soybean hulls, extruded soybean meal, corn grain, corn distillers dried grains with solubles, high protein distillers dried grains, and corn fermented protein were obtained from commercial industry sources. In addition, a complete diet consisting of corn (44.9%), solvent-extracted soybean meal (22.9%), corn distillers dried grains with solubles (29.7%), and minerals and vitamins (2.5%) was manufactured to simulate a typical commercial swine grower diet (24).

### 2.2 Chemical analysis of ingredients and complete feed

The chemical composition (i.e., moisture, crude protein, ether extract, neutral detergent fiber, and ash content) and water activity in each of the seven ingredients and complete feed were determined on day 0 for use in subsequent correlation analysis to explore potential associations between chemical composition of ingredients and virus inactivation rate. All ingredients and complete feed were subsampled, and samples were submitted to the University of Missouri Agricultural Experiment Station Chemical Laboratories (Columbia, MO, United States) for chemical analyses. Samples were analyzed using AOAC (25) procedures for crude protein (CP; Method 984.13), ether extract (EE; Method 920.39), ash (Method 942.05), and neutral detergent fiber (26). Dry matter content and water activity of samples were measured at the University of Minnesota. Water activity was assessed using a Decagon Pawkit (METER Group, Pullman, WA), and dry matter content was determined following the NFTA 2.2.2.5 method (27).

### 2.3 Surrogate virus assay and EhV stock

Access to ASFV is highly restricted and requires adhering to strict biosecurity protocols in government-approved high biosecurity research facilities (BSL-3). Therefore, we developed a surrogate virus assay (RISNA) using EhV to safely and accurately evaluate ASFV survival and mitigation in feed ingredients. The RISNA assay was used to assess EhV inactivation in feed ingredient and complete feed matrices for the storage stability and *in vitro* digestibility experiments. Previous studies have shown remarkable structural (28–30) and functional (15) similarities between ASFV and EhV, which makes EhV a suitable, safe surrogate for these types of experiments.

The EhV strain used in the current study (EhV-86) was provided by Dr. Martinez-Martinez laboratory (Bigelow – Laboratory for Ocean Sciences, East Boothbay, Maine). It was cultured in Alga-Gro® Seawater Medium (Carolina Biological Supply Company, Burlington, North Carolina) in a 15°C incubator until lysis occurred, which was observed after 4 days. The lysate was then filtered through a 0.45 μm filter (Nalgene™ Rapid-Flow™ Bottle Top Filters, ThermoFisher Scientific, MA, US) to remove cell debris. The filtered lysate was aliquoted, titered using flow cytometry, and stored in the dark at 4°C until use.Virus Viability Assay, DNA extraction and qPCR assay.

Platinum IV chloride (Pt_4_CL) was chosen as a suitable alternative reagent to replace PMAxx for assessing viable virus particles (31), and a pilot study was conducted to determine the optimal concentration of Pt_4_CL to ensure accurate estimation of the EhV viability. Results from this study showed that a dose of 1 mM Pt_4_CL provided a similar estimation of EhV viability as that achieved using 100 μM PMAxx (Supplementary Figure 1). Consequently, the viability assay was performed as described by Balestreri et al. (15), replacing PMAxx with 1 mM Pt4CL. Since PMAxx is a dye that requires light activation, the step of exposing samples to light for 30 min to cross-link PMAxx to DNA or RNA was also removed from the protocol. All other aspects of the assay procedure remained unchanged.

Viral DNA was isolated using automated extraction with the NucleoMag Virus kit (Macherey-Nagel, Düren, Germany) and a Magnetic Particle Processor (KingFisher Flex, Thermo Fisher Scientific, United States), following the manufacturer’s instructions. A sample volume of 200 μL with a 1 mM Pt4CL concentration was used, with an elution volume of 50 μL in molecular-grade water. Quantitative PCR was conducted using QuantiNova SYBR Green PCR kit (Qiagen, CA, United States) using the following conditions: 2 min at 95°C followed by 40 cycles of 5 s at 95°C and 10 s at 60°C (reaction mix components: SYBR Green PCR Master Mix, primer pair, molecular grade water, and 1 μL DNA template). The PCR assay was conducted using a QuantStudio 3 Real-Time PCR (Applied Biosystems, Thermo Fisher Scientific, Massachusetts, United States). Standards for the EhV qPCR assays were created as described by Balestreri et al. (15).

### 2.4 Experiment I—assessment of EhV viability under storage conditions

Samples of all feed ingredients and complete feed were inoculated with EhV on day 0 and stored at three different temperatures (4, 24 and 34°C) for up to 120 days to determine inactivation kinetics of EhV under simulated storage conditions. The storage temperatures were chosen based on historical temperature and relative humidity data for both land and oceanic segments of two 37-day transboundary shipping models for transporting feed ingredients from China and Europe to the United States (19). The range of temperatures for the China-Pacific-United States route was between 4 to 10°C (December – January conditions), and the range of temperatures for the Europe-Atlantic-USA was between 4 to 20°C (April–May conditions). In addition, a maximum temperature of 34°C was used to represent summer storage conditions in enclosed silos in the United States.

One set of triplicate samples were prepared by adding 1 g of each feed matrix to sterile 15 mL Falcon tubes (CorningTM Falcon 15 mL Conical Centrifuge Tubes, ThermoFisher Scientific, MA) and served as the positive controls (matrix or blank + EhV), while another set of triplicate samples of 1 g of each matrix served as negative controls (matrix or blank + medium). For the positive control samples, 200 μL of EhV-86 filtrate (1 × 10^8^ cells/mL) was added to each tube containing 1 g of each matrix, and 200 μL of AlgaGro® Seawater Medium (Carolina Biological Supplement Company, North Carolina, United States) was added to each 1 g matrix in tubes for the negative control samples. AlgaGro® Seawater Medium was used as a negative control because it is the medium in which the EhV virus grows and serves as the base solution for the positive control. In addition, 1 × 10^8^ cells/mL of stock virus was added to empty 15 mL tubes as the baseline for assessing the survival of EhV without the effect of the matrix under the simulated storage conditions. Both sets of triplicate samples of each feed matrix were placed in environmental chambers for each temperature (4, 24 and 34°C) and timepoint (1, 5, 60 and 120 days). Thus, a total of 648 samples were used in the experiment [9 treatments (8 matrices and a blank) × 6 samples per treatment (3 positive +3 negative controls) × 3 temperatures (4, 24 and 34°C) × 4 time points (1, 5, 60 and 120 days)]. At each time point, the two sets of triplicate treatments (positive and negative controls) for each temperature were removed from the environmental chambers to determine viable EhV concentrations.

### 2.5 Experiment II—assessment of EhV viability after *in vitro* stomach and small intestine digestion

An *in vitro* digestion assay (18) was modified to accurately estimate EhV viability on an experimental scale by determining the appropriate combination of virus inoculum concentration, matrix weight, and buffer volumes to ensure precise PCR measurement without causing dilution effects. This modified protocol was used to determine the fate of EhV during *in vitro* digestion of inoculated ingredients and complete feed after two enzymatic hydrolysis steps simulating stomach digestion (pepsin hydrolysis) and small intestine digestion (pancreatin hydrolysis).

Two sets of triplicate samples were prepared, which consisted of positive controls (matrix or blank + EhV) and negative controls (matrix or blank + medium). Additionally, the viability of EhV during the digestive process in the absence of a feed matrix was assessed, resulting in a total of nine treatments (eight matrices and one blank). All feed matrices were ground to pass a 1 mm mesh screen before undergoing *in vitro* pepsin and pancreatin hydrolysis. Approximately 100 ± 5 mg of each sample was weighed into sterile 15 mL tubes (Corning™ Falcon 15 mL Conical Centrifuge Tubes, ThermoFisher Scientific, MA). For the positive control samples, 2 mL of EhV-86 filtrate (1 × 10^8^ cells/mL) was added to each tube, and for the negative control samples, 2 mL of AlgaGro® Seawater Medium (Carolina Biological Supplement Company, North Carolina, United States) was added to each tube. Next, 6 mL of a previously prepared pepsin solution, as described by Huang et al. (18), was immediately added to each tube, and the tubes were placed in a water bath at 39 ± 0.5°C for 2 h under gentle agitation. At the end of the 2 h incubation period, samples were centrifuged at 4,700 rpm for 10 min. The concentration of viable EhV was then measured and calculated as previously described by Balestreri et al. (15). Immediately after the 2 h incubation period, the same samples were used to determine the effect of pancreatic hydrolysis by adding 2.5 mL of a previously prepared pancreatin solution (18) to each tube, placing them in a water bath at 39 ± 0.5°C for 4 h under gentle agitation, and determining the concentration of viable EhV as previously described by Balestreri et al. (15).

### 2.6 Experiment III—assessment of EhV viability after *in vitro* large intestine fermentation

Residues from enzymatic hydrolysis of each feed matrix without EhV inoculation were used to determine EhV viability during *in vitro* large intestine fermentation using procedures described by Huang et al. (18). Briefly, about 100 ± 5 mg of each hydrolyzed residue was weighed in a 125 mL serum bottle with rubber stoppers which contained 10 mL buffer solution with 5% fecal inoculum. The protocol for preparing the fecal inoculum was previously described by Huang et al. (18). The viability of EhV during the fermentation process was assessed in the absence of a feed matrix, in the fecal inoculum (fermentation buffer solution treatment), and in the EhV inoculated feed matrix residues, for a total of 10 treatments (eight feed matrices, fermentation buffer, and fecal inoculum). For each treatment, two sets of triplicates were used that consisted of a set of positive controls (matrix or solutions + EhV) and a set of negative controls (matrix or solutions + seawater medium). Immediately after adding the EhV and the fecal inoculum or the buffer solution, the bottles were placed in a water bath at 39 ± 0.5°C for 24 h. At the end of the 24 h incubation period, 200 μL samples were collected from each tube for the assessment of EhV viability as previously described in Balestreri et al. (15).

### 2.7 Statistical analysis

A simple linear regression analysis was used to analyze EhV viability data from Experiment I. In Experiment II, an ANOVA was used to assess differences in EhV viability during stomach and small intestine digestion among various feed matrices, and an independent two-sample t-test was used to determine if there were significant differences in EhV viability between the stomach-only and the stomach + small intestine digestion processes. Similarly, data analysis from Experiment III involved an ANOVA to evaluate differences in EhV viability during large intestine fermentation. A post-hoc Tukey’s test was used to assess differences between treatments in Experiments II and III. Experimental error associated with the viability PCR method was estimated to be in the ±1 log reduction range, which was determined empirically by analyzing multiple replicates of samples with known viral concentrations. Given that a single data point is derived from a PCR amplification plot (i.e., known as the threshold cycles reported as CT or Cq values), and that the error in CT over an exponential phase of amplification (where CT values are taken) is equivalent to doubling events, a 3-point difference in a CT value is approximately equivalent to a 10-fold change in the quantity of viral genetic material. This means that a differences in a single CT value between biological replicates can have an error greater than 30% entirely due to cycling inefficiencies, which can occur from pipetting errors, properties of the polymerase, or the characteristics of a given matrix from where the virus was extracted. Responses falling within this threshold were considered negligible and treated as zero for data analysis purposes, irrespective of the statistical methodology employed. All statistical tests were conducted at a significance level of *p* < 0.05. Data visualization and statistical analyses were conducted using RStudio (version 2024.04.1) and R (version 4.2.2). The R package of dplyr (version 1.1.2) was used for data manipulation, ggplot2 (version 3.5.1) was used for data visualization, and emmeans (version 1.8.6) and multcomp (version 1.4.25) were used for contrasts and multiple comparisons.

## 3 Results

### 3.1 Experiment I—assessment of EhV viability under storage conditions

**Figure 1:**
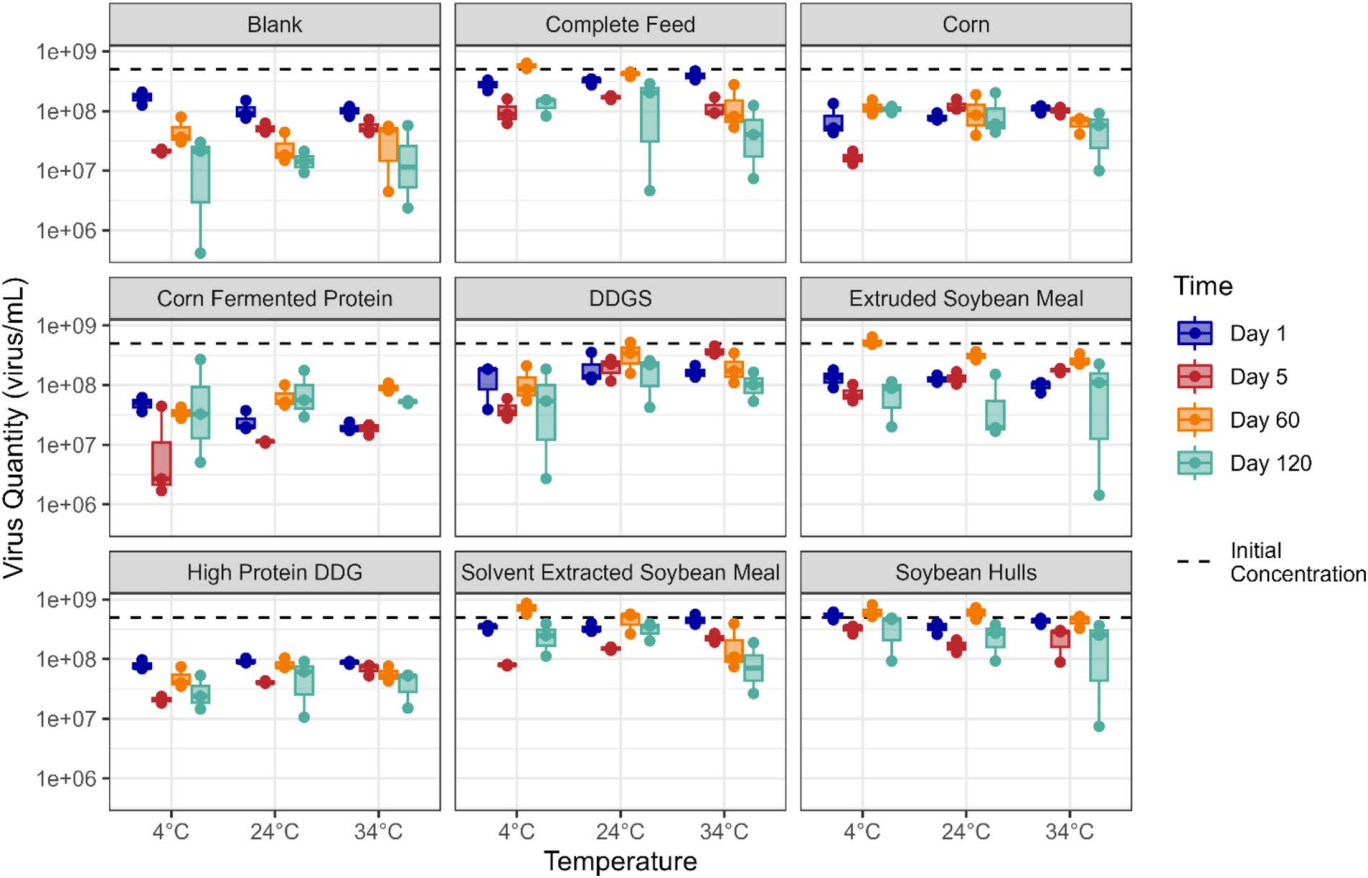
Boxplot illustrating the quantity (virus/mL) of viable EhV in different feed matrixes at different temperatures (x-axis) and timepoints (colors indicate days post-inoculation). The red dashed line denotes the EhV virus quantity at day 0.

Viability of EhV was determined in all corn- and soybean-based ingredient and complete feed samples stored at 4°C, 24°C, and 34°C on days 1, 5, 60, and 120 post-inoculation (Figure 1). Statistically significant linear reductions were observed for viable EhV concentrations across time and temperature conditions evaluated in this study. However, despite the statistical significance of these responses, the differences observed did not exceed the margin of error for the PCR viability assay, which was estimated to be ±1 log concentration across these time and temperature conditions (Table 1).

**Table 1:** Effect of time and temperature on viable EhV concentration difference.

Feed Matrix	Effect of temperature (*Δ* Temperature^1^) at				Effect of time (Δ Time^2^) at		
	Day 1	Day 5	Day 60	Day 120	4°C	24°C	34°C
Logarithmic concentration difference							
Blank	−0.24*	0.42*	−0.31	0.32	−0.95	−0.75*	−0.86*
Complete Feed	0.14*	0.12	−0.66*	−0.56	−0.02	−0.48	−0.81*
Corn	0.20	0.86*	−0.26	−0.41	0.55*	−0.08	−0.46*
Corn Fermented Protein	−0.40*	0.49	0.42*	0.20	0.33	0.66*	0.53*
DDGS	0.19	0.99*	0.34	0.57	−0.30	−0.10	−0.39*
Extruded Soybean Meal	−0.11	0.39*	−0.32*	−0.26	−0.08	−0.44	−0.50
High Protein DDG	0.05	0.50*	0.11	0.14	−0.18	−0.17	−0.35*
Solvent Extracted Soybean Meal	0.10	0.45*	−0.64*	−0.32	0.21	0.17	−0.68*
Soybean Hulls	−0.12	−0.24	−0.13	−0.45	−0.14	0.02	−0.47

1 Viable EhV concentration at 4°C minus viable EhV concentration at 34°C; calculated using linear regression.

2 Viable EhV concentration at d 1 minus viable EhV concentration at d 120; calculated using linear regression.

*Linear effect (*p* < 0.05).

**Figure 2:**
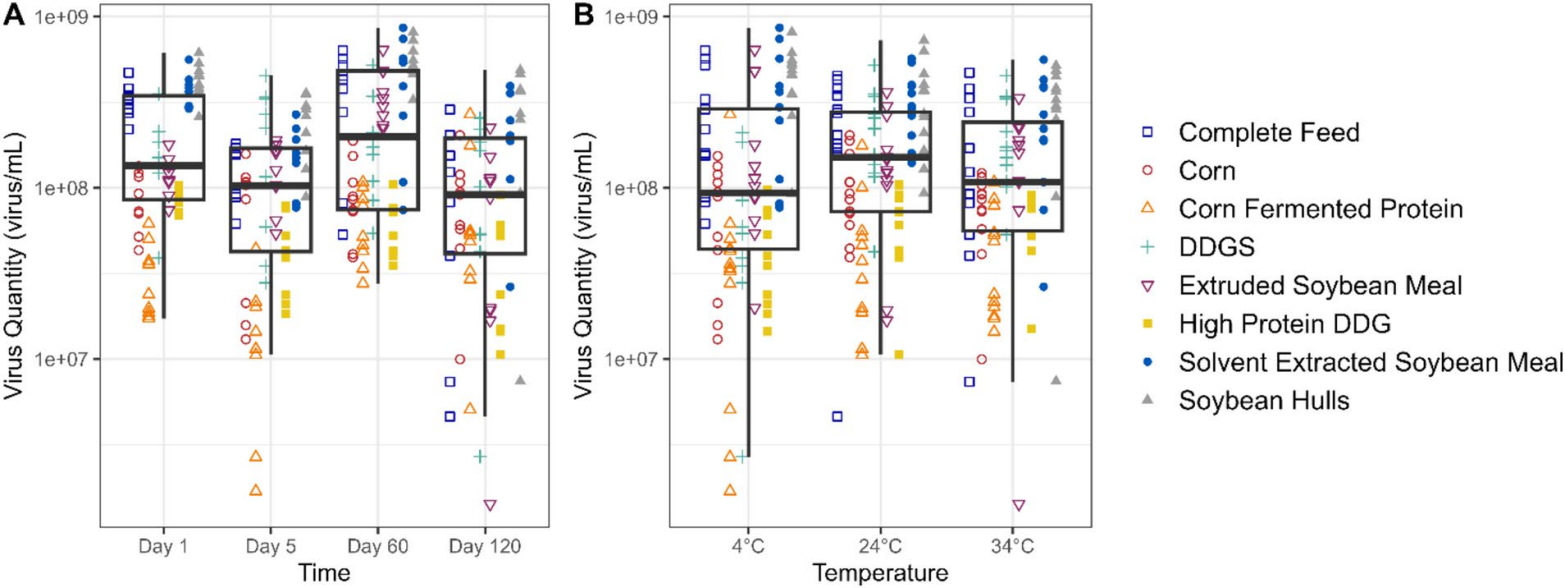
Boxplot depicting the quantity (virus/mL) of viable EhV in different feed matrixes at different storage time points **(A)** and temperatures **(B)**.

Figure 2A presents the data on viable EhV across a 120-day storage period. Although a statistically significant trend was observed in viable EhV across all matrices combined (*p* = 0.006), the reduction in viable EhV concentration was 0.2 log, which falls within the margin of error for the PCR viability assay, and therefore the linear relationship is not depicted in the plot. Figure 2B illustrates the relationship between EhV viability and temperature, showing no significant differences in EhV inactivation across temperatures (*p* = 0.483) for all matrices combined. Thus, no reductions in EhV viability exceeding a 1 log concentration were detected at any temperature or for any matrix throughout the 120-day experimental period.

**Figure 3:**
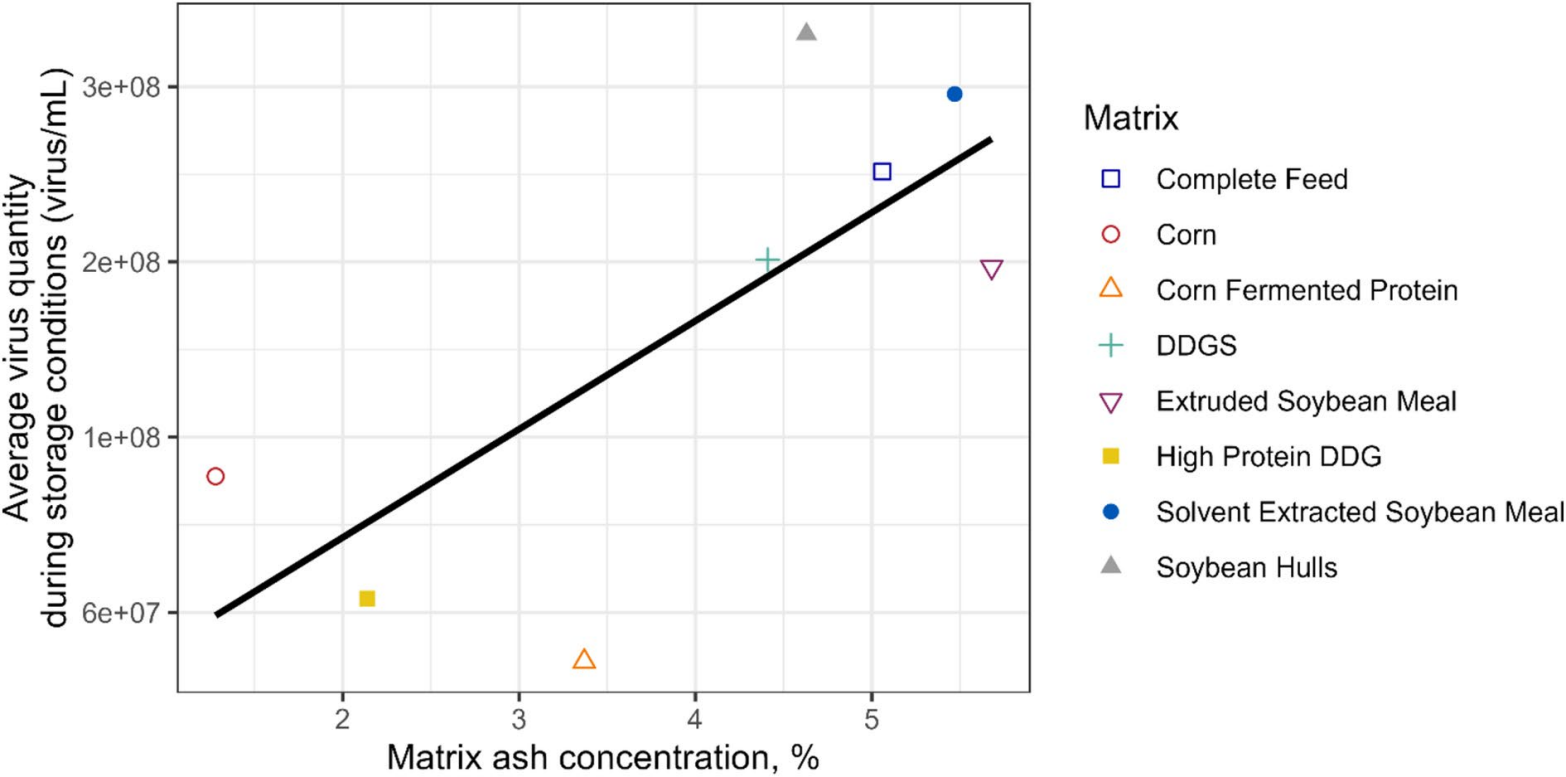
A linear association (*p* = 0.034) was observed between the feed matrix ash concentration and the average viable EhV quantity during the storage conditions considering all time points and temperatures combined.

The chemical composition and water activity of the feed matrices evaluated in this study were determined (Table 2). There were wide ranges in CP (7.8–49.3%), EE (0.8–7.27%), ash (1.28–5.69%), NDF (8.7–58.6%), and water activity (0.32–0.70 a_w_). Ash concentration of the corn- and soybean-based feed ingredients evaluated was linearly associated (*p* = 0.034) with average EhV concentration during all storage time points and temperatures combined (Figure 3). However, the predicted potential protective effect of ash content in feed ingredients on EhV did not exceed the calculated experimental error of ±1 log. No other significant correlations were observed regarding the chemical composition of the feed matrices and viable EhV concentrations. Because no EhV inactivation was observed under any of the tested storage conditions (4°C, 24°C, and 34°C at 1, 5, 60, and 120 days of storage), no correlations could be calculated between chemical composition, water activity, and EhV inactivation.

**Table 2:** Nutritional composition of feed ingredients and complete feed.

Feed Matrix	Nutritional component, %					Water activity a_w_
	Dry matter	Crude protein	Ether extract	Ash	Neutral detergent fiber	
Complete Feed	87.4	21.6	2.47	5.06	17.8	0.70
Corn	88.5	7.83	3.04	1.28	12.8	0.59
Corn Fermented Protein	93.1	49.2	3.55	3.37	42.7	0.53
DDGS	87.6	30.7	6.77	4.41	30.3	0.62
Extruded Soybean Meal	95.6	44.7	7.27	5.68	11.4	0.32
High Protein DDG	93.4	49.3	6.99	2.14	36.6	0.51
Solvent Extracted Soybean Meal	87.8	47.2	1.17	5.47	8.66	0.67
Soybean Hulls	90.4	9.89	0.8	4.63	58.6	0.56

### 3.2 Experiment II—assessment of EhV viability after *in vitro* stomach and small intestine digestion

**Figure 4:**
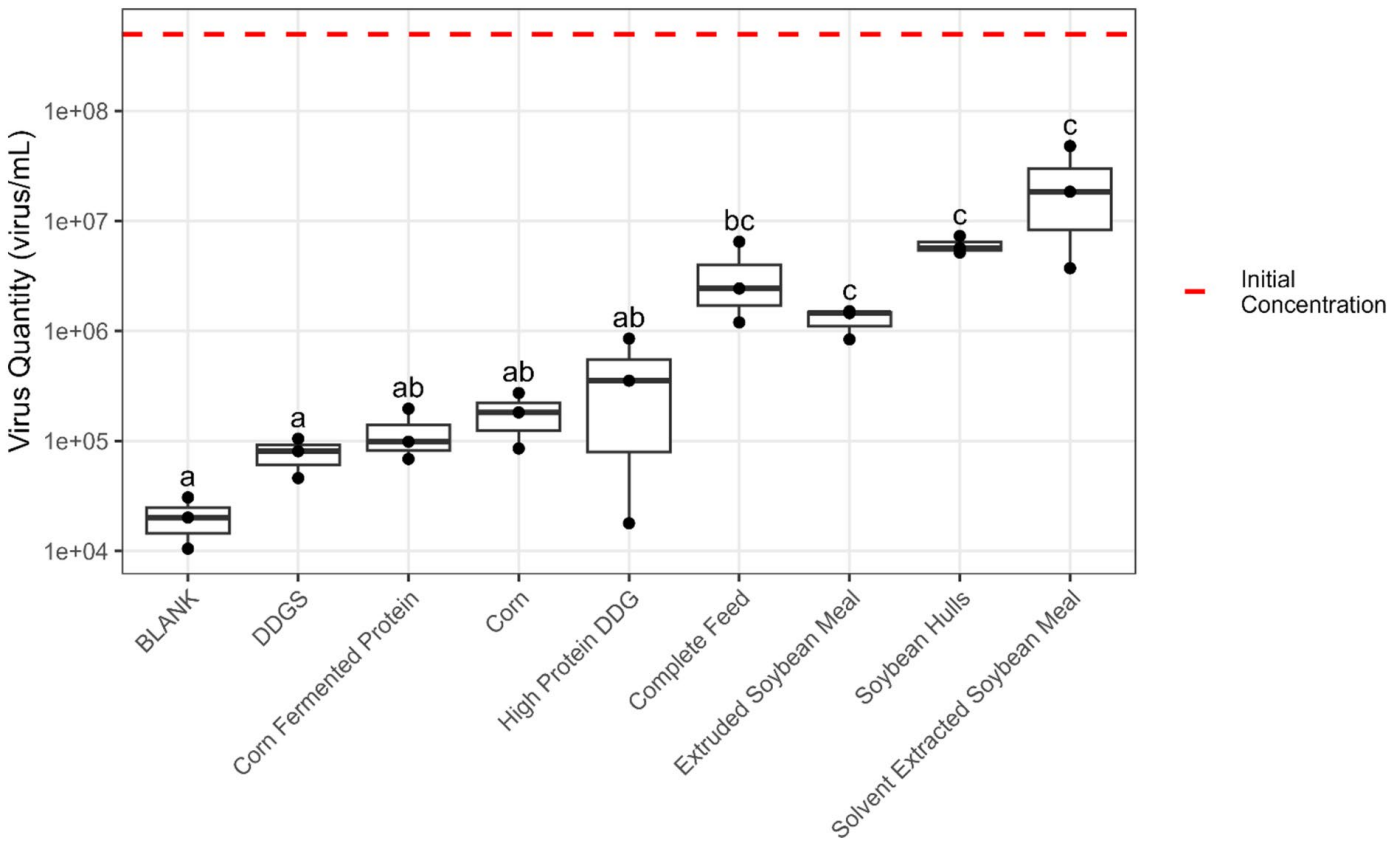
Boxplot of the quantity of viable EhV (virus/mL) after the 6 h *in vitro* digestion process using pepsin + pancreatin to simulate stomach and small intestine digestion. The red dashed line represents the initial EhV virus concentration. Matrices without a common letter are significantly different (*p* < 0.05) according to the Tukey test.

Pepsin + pancreatin digestion (simulating stomach and small intestine conditions) resulted in an average reduction of 2.8 log units in EhV viability among all feed matrices, but soybean-based ingredients had a greater protective effect on virus viability than corn and corn co-products (Figure 4). However, when comparing the virus stability in stomach-only (pepsin hydrolysis) with combined stomach + small intestine (pepsin + pancreatin hydrolysis) conditions, no differences exceeding the estimated experimental error of the viability PCR method (±1 log) were observed.

### 3.3 Experiment III—assessment of EhV viability after *in vitro* large intestine fermentation

**Figure 5:**
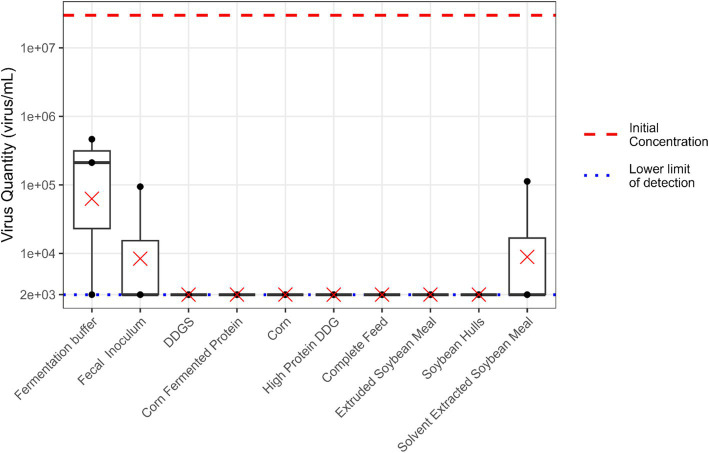
Boxplot illustrating the quantity of viable EhV (virus/mL) during 24 h *in vitro* large intestine fermentation. The red dashed line represents the initial EhV virus concentration. The blue dotted line indicates the lower limit of detection for EhV viability. Mean values are denoted by “×” symbols for each feed matrix.

An average reduction of 2.68 log units in viable EhV concentrations was observed in the buffer solution with no feed matrix or fecal inoculum (Figure 5). Additionally, fermentation with buffer and fecal inoculum but without predigested substrate resulted in an average reduction of 3.8 log units in viable EhV concentration over a 24 h time period. The average viable EhV concentration in solvent extracted soybean meal was reduced by 3.53 log units during *in vitro* fermentation, while all other feed matrices had an average reduction of 4.1 log units during the 24 h fermentation period. However, it should be noted that these calculated reductions are minimum values, and the actual virus inactivation may have been greater. Exact reductions could not be determined due to the lower limit of detection (LOD) in the experiment. No statistically significant differences were observed among treatments in EhV viability during the *in vitro* fermentation process (*p* = 0.097).

## 4 Discussion

No system for monitoring or standardized testing of the potential presence and concentration of ASFV in feed ingredients exists in global or domestic feed supply chains. As a result, very little is known about the likelihood of ASFV transmission in feed. In fact, a recent risk assessment on ASFV transmission conducted by Bergmann et al. (32) did not include feed as a potential factor. Among the limited number of other risk assessments conducted for feed ingredients, two have been qualitative without indicating that there is a high degree of uncertainty (33, 34), one was quantitative but only included imported corn and soybean meal from ASFV-positive countries into the United States (12), and one was quantitative but focused only on disease status of the country of origin (11). Because of the high uncertainty of knowing whether a feed ingredient imported from an ASFV-positive country is contaminated, and the need for high biosecurity due to the lack of commercially available preventive vaccines, use of extended storage times has become a popular mitigation approach for partial ASFV inactivation if it is present. Overall, current evidence of ASFV transmission through feed under field conditions is circumstantial. Confronted with no monitoring systems, food safety professionals may assume potential virus contamination and consequently use a microbiological risk assessment model as a prudent approach to address the issue (35). A microbiological risk assessment can be used to calculate scenarios for initial virus concentration and the appropriate amount of virus inactivation for the performance objective. An example of using a performance objective to assess risk of ASFV in spray dried porcine plasma was recently published (36). However, very few studies report the d-values of virus inactivation and likelihood of contamination in the scientific literature.

### 4.1 Experiment I—assessment of EhV viability under storage conditions

Although statistically significant linear reductions were observed for viable EhV concentrations across time and temperature conditions evaluated in this study, the differences observed did not exceed the margin of error for the PCR viability assay, which was estimated to be ±1 log concentration across these time and temperature conditions. As a result, it is uncertain whether the differences observed represent actual virus inactivation or if they are artifacts of experimental error. Although an initial reduction of 0.5 log in viable EhV concentration occurred between day 1 and day 5, when considering the viable EhV concentrations at day 60 and day 120, this reduction appears to be related to experimental error rather than virus inactivation.

No reductions in EhV viability exceeding a 1 log concentration were detected for any matrix at any temperature throughout the 120-day experimental period because the experimental error was within the range of 1 log concentration. Therefore, these results indicate that storing corn- and soybean-based feed ingredients and completed feed inoculated with EhV at 4°C, 24°C, or 34°C for up to 120 days had a negligible effect on inactivation of EhV in all feed ingredients and complete feed matrices evaluated. Because EhV is an ASFV-like virus, these results indicate that ASFV can maintain viability in various feed matrices during long-term storage and suggest that extending storage time alone may not be an effective mitigation practice for ASFV.

Only a few studies have been conducted to determine the effect of storage time and temperature on ASFV survival in feed ingredients and complete feed. Unfortunately, the effectiveness of extended storage is difficult to interpret because of the analytical methods used to determine ASFV concentration. Stoian et al. (20) reported half-life values for conventional and organic soybean meal, complete feed, pet foods, choline, and pork sausage casings that were experimentally inoculated with 10^5^ TCID_50_, which ranged from 9.6 (conventional soybean meal) to 14.2 (complete feed) days during a simulated 30-day transoceanic shipment at an average temperature of 12.3°C and average relative humidity of 74.1%. Half-life is an estimate of the amount of time it takes for half of the virus to be inactivated but does not indicate viability or infectivity of the virus. Fischer et al. (37) evaluated the effects of inoculating spray-dried porcine plasma with 10^6^ HAD_50_/mL and storing it for up to 35 days at 4°C and 21°C. For this feed matrix, the ASFV concentration was reduced by >5.7 log after 2 weeks of storage at 21°C. Although the HAD_50_ assay is used as a method for estimating the infectivity of ASFV, pigs can become infected after exposure to only a few virus particles while others may require a concentration of 10^7^ HAD_50_ for infection. Furthermore, the HAD_50_ assay only measures the viruses that can attach to red blood cells, but viruses that lose this HA phenotype are also infectious. Therefore, the HAD_50_ method is not a definitive measure of ASFV infectivity. For example, Niederwerder et al. (22) reported that although a low dose of 10^2^ HAD_50_ did not cause ASFV infection, a moderate (10^4^) dose was sufficient to cause infection. In another study, Niederwerder et al. (21) determined the stability of an ASFV Georgia 2007 isolate in complete feed, soybean meal, and ground corn cobs when exposed to 4°C, 20°C, and 35°C for up to 365 days using qPCR, virus isolation, and swine bioassays. Soybean meal required the longest amount of time for reduction in ASFV infectivity followed by complete feed and corn cob particles, which led to the recommendation that ASFV-contaminated feed be stored for >112 days at 4°C, >21 days at 20°C, and > 7 days at 35°C. These recommendations are not consistent with those observed in the current study.

Two other studies reported conflicting results when various types of feed ingredients were exposed to higher temperatures (>40°C) for shorter periods of time (< 2 h). Fischer et al. (38) determined ASFV concentrations in wheat, barley, rye, triticale, corn, and peas inoculated with ASFV-infected blood (10^6^ HAD_50_/mL) and reported that ASFV was detected in all samples by PCR when dried at 20°C for 2 h and incubated for 1 h at 75°C, but no infectivity, as measured by HAD_50_ and virus isolation, was observed after 2 h of storage at 20°C. Songkasupa et al. (39) used HAD_50_ to quantify ASFV concentrations, calculate D values (time required to reduce ASFV by 1 log at a specific temperature), and develop models to predict ASFV inactivation in corn, soybean meal, and meat and bone meal when exposed to temperatures of 60, 70, 80, and 90°C for 20 min. They observed no differences in D values and heat resistance among ingredients. Furthermore, exposure of feed ingredients to high temperatures for longer periods, such as the multiple weeks spanned by the current study, results in degradation of lipids, proteins, and vitamins (13). Therefore, holding feed ingredients at high temperatures during long storage periods is not a feasible approach for inactivating ASFV.

Although the ash concentration of the corn- and soybean-based feed ingredients was linearly associated (*p* = 0.034) with average EhV concentration during all storage time points and temperatures combined, the predicted potential protective effect of ash content in feed ingredients on EhV did not exceed the calculated experimental error of ±1 log which suggests that the observed effect may be attributable to experimental error rather than a true protective effect. No other significant responses were observed regarding the chemical composition of the feed matrices and viable EhV concentrations. Physical characteristics and chemical composition of feed matrices are likely to play an important role in EhV and ASFV survival and inactivation, but very little is known about these potential effects. A moderate correlation has been observed between moisture concentration of feed ingredients and increased survival of porcine delta coronavirus (*r* = 0.48) and transmissible gastroenteritis virus (40). Water activity of food matrices is a good predictor of thermal resistance of bacterial pathogens in foods (41), but is rarely determined in feed ingredients. A previous study showed that water activity was greater in soybean meal, barley, rapeseed cake, and corn that was milled to a coarse particle size compared with fine particle size, with coarse milled soybean meal having the greatest water activity (42). Solvent extracted soybean meal (0.67 a_w_) and complete feed (0.70 a_w_) had the greatest water activity among feed matrices evaluated in the current study. Other compounds such as isoflavones and saponins in soybean meal (43) and copper and zinc (44–49) have been shown to have antiviral and antimicrobial properties. The addition of sodium chloride has been shown to be effective in partially inactivating porcine delta coronavirus (50) and porcine epidemic diarrhea virus (51) in complete feed. However, because no EhV inactivation was observed under any time and temperature conditions evaluated in this study, no correlations between composition, water activity, and EhV inactivation could be estimated. Nonetheless, this phenomenon warrants further investigation because it has the potential to explain unknown dynamics of ASFV infection from feed consumption.

### 4.2 Experiment II—assessment of EhV viability after *in vitro* stomach and small intestine digestion

Although there is no direct evidence indicating that feeding naturally contaminated feed to pigs causes disease under field conditions, Oļševskis et al. (52) suggested that feeding swill and potentially contaminated fresh grass or crops were probable causes of ASFV outbreaks on some swine farms in Latvia but provided no definitive evidence for this potential route of transmission. Similarly, Wen et al. (53) was unable to isolate live ASFV from dried blood meal samples used in swine feed, but inferred it was a “highly likely” source for the spread of ASFV in China. Zhai et al. (54) also suggested that feed was a cause of ASFV transmission in China despite providing any quantitative evidence. Likewise, Gebhardt et al. (55) collected 54 samples of complete feed and feed ingredients from a feed mill serving multiple internal and external swine production sites that were contaminated with ASFV, but none of the samples tested positive for ASFV using a PCR assay. However, these researchers noted that all feed manufactured for internal use contained a commercial formaldehyde-based feed additive used at the recommended dose. A commercially available formaldehyde product is approved for use in Salmonella control in the United States but not for ASFV. Formaldehyde may inactivate ASFV by inducing DNA damage, cell damage, and interference with virus replication. These effects are dependent on concentration which is best described as the decimal-concentration [d-value; (56)]. It is also important to note that the DNA damage effects of formaldehyde-based products can still render the DNA detectable by PCR (7). Therefore, the negative PCR result observed by Gerhardt et al. may indicate a genuine absence of ASFV in the samples. Unger et al. (57) showed a correlation between the frequency of ASFV-infected pigs and their proximity to bodies of water but provided no direct evidence to indicate ASFV-contaminated water was the cause of infection. However, studies have shown that ASFV can survive and remain infectious in experimentally inoculated feed for up to 365 days (21) and for up to 42 days in river water at 4°C (58).

Despite the lack of direct evidence for transmission of ASFV through feed under field conditions, Niederwerder et al. (22) showed that feed and water can be routes of ASFV transmission by determining the minimum infectious dose of 10^4^ TCID_50_ in feed and 10^0^ TCID_50_ in liquid. However, Blázquez et al. (23) reported that the minimum infectious dose of ASFV is greater than 10^5^ because feeding diets inoculated with 10^5^ TCID_50_ of ASFV in liquid plasma for 14 consecutive days failed to cause disease.

Findings from the current study suggest that all feed matrices provided some level of protection to EhV. However, soybean-based ingredients and complete feed exhibited significantly greater protective effects on virus viability compared with responses in corn and corn co-products. When comparing the virus stability in stomach-only (pepsin hydrolysis) with combined stomach + small intestine (pepsin + pancreatin hydrolysis) conditions, no differences exceeding the estimated experimental error of the viability PCR method (±1 log) were observed. For Experiments II and III, the viability PCR method had an LOD of 2.5 × 10^3^ viral particles due to inherent dilution effects and practical constraints preventing further scaling of the experiment. Therefore, viable EhV concentrations observed as numerically zero correspond to levels at or below the LOD, which is equivalent to a 3.4 log concentration. Matrices yielding negative results (zero viable EhV concentration) indicated a reduction in viral concentrations of at least the reported logarithmic units during the 24 h *in vitro* fermentation process.

### 4.3 Experiment III—assessment of EhV viability after *in vitro* large intestine fermentation

No differences were observed among treatments in EhV viability during the *in vitro* fermentation process. However, exact reductions in EhV concentrations could not be determined due to the low LOD of the viability PCR method of 2.5 × 10^3^ viral particles due to inherent dilution effects and practical constraints preventing further scaling of the experiment. Therefore, viable EhV concentrations observed as numerically zero correspond to levels at or below the LOD, which is equivalent to a 3.4 log concentration. Matrices yielding negative results (zero viable EhV concentration) indicated a reduction in viral concentrations of at least the reported logarithmic units during the 24 h *in vitro* fermentation process.

Based on an average reduction of 2.8 log units in EhV viability observed during stomach + small intestine digestion across all matrices, and an additional 3.8 log reduction during large intestine fermentation across all matrices, we estimate an average total reduction of about 6.7 log units during the entire total tract *in vitro* digestion and fermentation process. This reduction in average EhV viability ranged from 5.32 log units for solvent-extracted soybean meal to 7.47 log units for corn, which may be greater due to the LOD of the experiment. These results may explain differences in infectious doses among feed ingredient matrices reported by Niederwerder et al. (22) and Blázquez et al. (23). Differences in timing of virus release from feed ingredient matrices during the hydrolysis portion of the digestion process may partially explain the inconsistencies in ASFV infection when pigs consume infectious doses of contaminated feed.

## 5 Conclusion

Using EhV as a safe and suitable ASFV-like surrogate virus enables the conducting of challenging experiments to begin understanding the dynamics of ASFV survival and inactivation in various types of feed matrices under various conditions. Unlike results from previous studies, our results showed no appreciable viable virus inactivation in either corn- or soybean-based feed ingredients and complete feed when inoculated with 10^8^ EhV/mL and stored at 4°C, 24°C, or 34°C for up to 120 days. Therefore, the use of extended storage time up to 120 days does not appear to be an effective mitigation practice against ASFV. We are also the first to report that between 5 to more than 7 log (99.999 to 99.999%) reductions in EhV in various feed matrices occur during the entire *in vitro* digestion and fermentation process. These reductions in EhV viability during the digestion process may be correlated with the ash concentrations in feed ingredient matrices, which may potentially explain inconsistencies in ASFV infection when pigs consume infectious doses of contaminated feed. Results from this initial study provided interesting new insights regarding the resiliency of EhV as a surrogate for ASFV in common feed matrices and simulated swine digestion and fermentation processes that will need to be confirmed by subsequent studies.

## Data Availability

The original contributions presented in the study are included in the article/Supplementary material, further inquiries can be directed to the corresponding authors.
