# Supplementary material for: Stability of a surrogate African swine fever-like algal virus in corn- and soybean-based feed ingredients during extended storage and in vitro digestion processes
Source: Front Vet Sci. 2024 Nov 27;11:1498977. doi: 10.3389/fvets.2024.1498977 (PMC11631853; doi:10.3389/fvets.2024.1498977)
Supplement: Supplementary file 1 [file Image_1.pdf]

Supplementary Material

1.1 Supplementary Figures

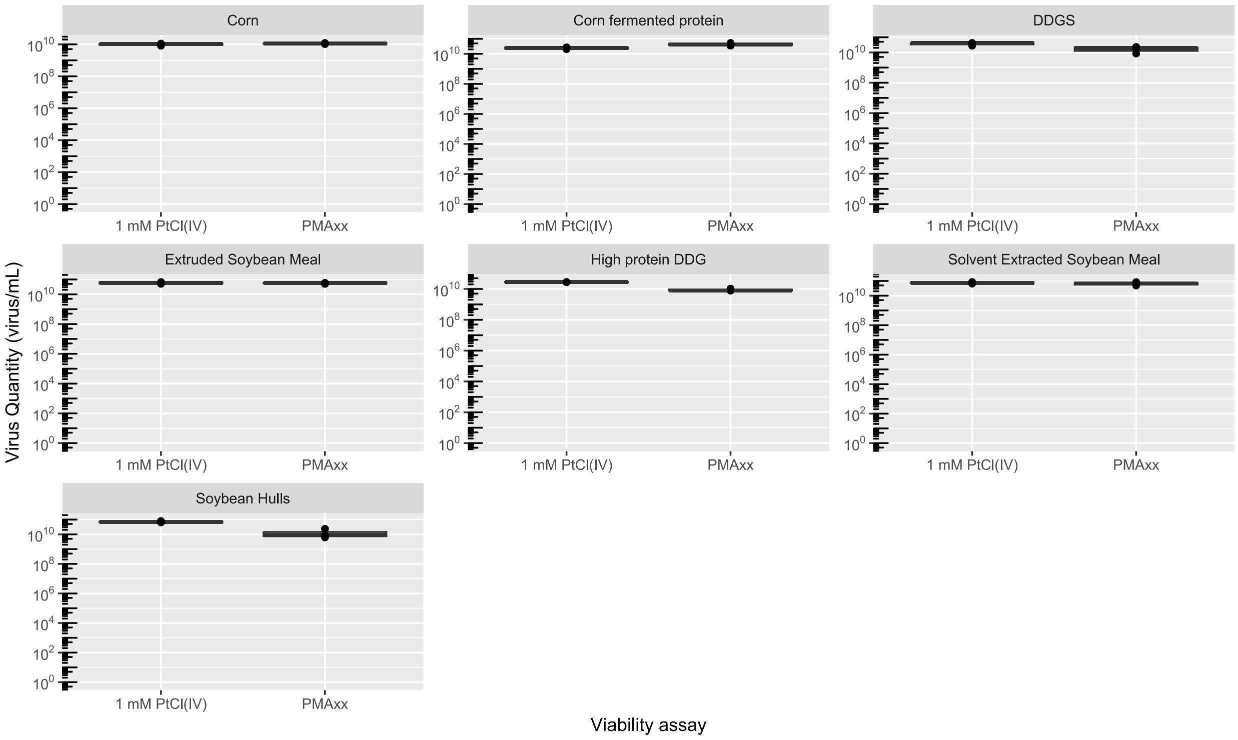

Supplementary Figure 1. Comparison of EhV viability estimated using either 1 mM platinum chloride ($PtCl_4$) or 100 µM PMAxx.
